# Addressing genetic diversity and health inequities: RELIVAF’s proposal for Latin American pharmacogenomic guidelines

**DOI:** 10.3389/fphar.2026.1721828

**Published:** 2026-01-22

**Authors:** Luis A. Quiñones, Matías F. Martínez, Rodrigo Vargas, Leslie C. Cerpa, Andrés López-Cortés, Farith González-Martínez, Dora Fonseca, Jorge Duconge, Nelson M. Varela, Ismael Lares-Asseff, María Ana Redal, Patricia Esperón, Fabricio Ríos-Santos, Ana Lucía Rendón, Roberto Serrano, Elizabeth Ortega, Luis Sullón-Dextre, Enrique Sanchez, María Laura Arias, Diadelis Remírez, Alexis Morales

**Affiliations:** 1 Laboratory of Chemical Carcinogenesis and Pharmacogenetics (CQF), Department of Basic and Clinical Oncology, Faculty of Medicine, University of Chile, Santiago, Chile; 2 Department of Pharmaceutical Sciences and Technology, Faculty of Chemical and Pharmaceutical Sciences, University of Chile, Santiago, Chile; 3 Center for Cancer Prevention and Control (CECAN), Santiago, Chile; 4 Laboratory of Advanced Research in Personalized Medicine, Department of Pharmaceutical Sciences and Technology, Faculty of Chemical and Pharmaceutical Sciences, University of Chile, Santiago, Chile; 5 Department of Molecular Biology, Galileo University, Guatemala, Guatemala; 6 Cancer Research Group (CRG), Faculty of Medicine, Universidad de Las Américas, Quito, Ecuador; 7 Toxicology and Public Health Research Laboratory, Department of Research, Faculty of Dentistry, University of Cartagena, Cartagena, Colombia; 8 Universidad del Rosario. School of Medicine and Health Sciences. Center for Research in Genetics and Genomics (CIGGUR), Institute of Translational Medicine (IMT), Bogotá, Colombia; 9 Department of Pharmaceutical Sciences, School of Pharmacy, University of Puerto Rico-Medical Sciences Campus, San Juan, PR, United States; 10 Latin American Society of Pharmacogenomics and Personalized Medicine, Santiago, Chile; 11 Molecular Diagnostic Laboratory, Genetics Division. Faculty of Medicine, Hospital de Clínicas José de San Martín. University of Buenos Aires, Buenos Aires, Argentina; 12 Molecular Genetic Unit, School of Chemistry, Universidad de la República, Montevideo, Uruguay; 13 Department of Health. Faculty of Medicine, Federal University of Mato Grosso (UFMT), Cuiabá, Brazil; 14 Department of Pharmaceutical Technology, National Autonomous University of Honduras, Tegucigalpa, Honduras; 15 PhV Latam, San Salvador, El Salvador; 16 Colegio de Bioquímica y Farmacia de Tarija, Tarija, Bolivia; 17 Sociedad de Farmacología Molecular del Perú, Lima, Peru; 18 Vivian Pellas Hospital, Managua, Nicaragua; 19 Tropical Diseases Research Center and Microbiology Faculty, University of Costa Rica, San José, Costa Rica; 20 National Centre for Quality Control of Drugs, La Havana, Cuba; 21 Facultad de Farmacia, Universidad de Los Andes, Merida, Venezuela

**Keywords:** clinical guidelines, diversity, Latin America, pharmacogenetics, pharmacogenomics

## Abstract

Latin America’s exceptional genetic diversity, shaped by centuries of admixture among Native American, European, and African ancestries, presents both challenges and opportunities for pharmacogenomic implementation. Current guidelines by CPIC and DPWG, though foundational, are largely based on European and East Asian data, limiting their applicability in highly admixed populations. This article presents the rationale and methodology of RELIVAF (Latin American Network for the Implementation and Validation of Pharmacogenomic Clinical Guidelines), which aims to produce region-specific recommendations aligned with local genetic profiles, healthcare systems, and regulatory landscapes. The framework integrates international standards with country- and ancestry-specific allele frequencies, effect sizes, drug availability, and implementation constraints. It also incorporates educational strategies to promote pharmacogenomic literacy among healthcare professionals. Three gene-drug pairs were prioritized for initial guideline development: *DPYD*-fluoropyrimidines, *TPMT/NUDT15*–thiopurines (paediatric ALL), and *CYP2C9/VKORC1*-coumarin anticoagulants (e.g., warfarin, acenocoumarol). Selection was based on clinical relevance, allele frequency variability, and potential public health impact. By leveraging regional data and collaborative expertise, RELIVAF aims to deliver actionable, equitable, and context-specific pharmacogenomic guidance, advancing precision medicine in Latin America and serving as a model for other underrepresented regions.

## Introduction

Ethnic disparities in drug response are well established. They stem from both socioeconomic inequalities and population-specific genetic variation, such as SNPs, insertions, deletions, and copy number variations in pharmacogenes (Ramamoorthy et al., 2022). These disparities are compounded by the underrepresentation of certain groups in genomic studies and clinical trials, limiting the generalizability and equity of precision medicine. Recent analyses by RELIVAF have underscored these gaps in Latin America and called for more inclusive research frameworks (Guerrero et al., 2018; Ramamoorthy et al., 2022; García-Cárdenas et al., 2024). Variants affecting pharmacokinetics and pharmacodynamics often follow ancestry-specific patterns shaped by evolutionary and sociocultural factors. Ethnicity is therefore a critical, but frequently underestimated, determinant of drug efficacy and toxicity (Homburger et al., 2015; Salzano and Sans, 2014; Ruiz-Linares et al., 2014).

Multiple examples illustrate this point. For instance, African Americans require higher doses of vitamin K antagonists (VKAs) to achieve therapeutic International Normalized Ratio (INR) levels compared to Asians, and they respond differently to beta-blockers than Caucasians (Johnson, 2008). These differences are largely attributable to allelic heterogeneity, as well as variations in allele frequency and effect size in genes such as *CYP2C9*, *VKORC1, CYP4F2, and rs12777823*, particularly when SNPs exhibit greater frequency differentiation across ancestral groups. Decreased-function variants in *CYP2C9* display marked ancestry-specific frequencies. While CYP2C9*2 and 3 alleles are more common in Europeans (∼7–10%) and infrequent among individuals of African ancestry (∼1–2%), other reduced-function alleles such as CYP2C9*8 occur at substantially higher frequencies in African Americans (∼6%). In this context, the incorporation of polymorphisms such as *CYP2C9* *8, *5, 6, and 11 into warfarin dosing algorithms has enabled the reclassification of metabolizer phenotypes in approximately 15% of African American individuals (Suarez-Kurtz, 2011). This highlights the risk that ancestry-enriched alleles may be underrepresented in genotyping panels commonly used across Latin America, where certain variants may remain untested despite potential functional impact.

Furthermore, the lack of a significant effect of CYP2C19*2 in predicting poor response to clopidogrel (measured as residual on-treatment platelet reactivity) among Latinx populations highlights their unique genetic landscape and challenges the relevance of this risk allele in explaining clopidogrel effectiveness in individuals of non-European ancestry (Yang et al., 2025; Echeverría et al., 2024). These groundbreaking findings shed light on the role of locus-specific ancestry in pharmacogenetic studies involving admixed populations. The frequency of the CYP2C19*2 (rs4244285) allele among Latinx individuals (12.3%) is significantly lower than that observed in Europeans (14.6%), Asians (31.3%), and African Americans (17.5%) (Melin et al., 2019).

Another example is 6-mercaptopurine (6-MP), a chemotherapeutic agent metabolized by thiopurine S-methyltransferase (TPMT). *TPMT* polymorphisms occur in ∼10% of Caucasians but are rare (<1%) in South and East Asians (Collie-Duguid et al., 1999; Kodidela et al., 2020). Data for Latin American populations remains incomplete.

*CYP2D6* polymorphisms also show wide interethnic variation: approximately 30% of individuals in the U.S. (Caucasians and African Americans) carry reduced-function alleles, compared to 2%–5% in Vietnamese and Gambian populations. These differences are also evident in the frequency of *CYP2D6* alleles associated with ultrarapid metabolizer (UM) phenotypes, which are more prevalent in Latin American populations (with values reaching up to 9.7%) compared to European populations (∼2%). This disparity may be attributed to the greater African genetic contribution in Latinx populations, as individuals of African ancestry exhibit the highest profile of UM (20%–29%) (Ramírez et al., 2019; Koopmans et al., 2021; Ramírez et al., 2023). Latin American frequencies require further specification but are expected to differ significantly due to high admixture levels, population stratification, adaptive introgression by ancestry-enriched SNPs, differential linkage disequilibrium (LD) patterns, and the resulting genetic heterogeneity.

## Genetic diversity and pharmacogenomics in Latin America: a unique opportunity for precision medicine

Encompassing approximately 640 million inhabitants across 33 countries, Latin America harbours one of the most recently and extensively admixed populations worldwide, resulting from historical admixture among the European, African, and Native American ancestries (Asia). This diversity is distributed heterogeneously across the region: The Amerindian ancestry predominates in Mexico and Guatemala, the African heritage is most prevalent in the Caribbean and Brazil, and the European ancestry has a greater presence in South American countries such as Argentina, Brazil, Chile, and Uruguay (Homburger et al., 2015; STATISTA, 2025).

**FIGURE 1:**
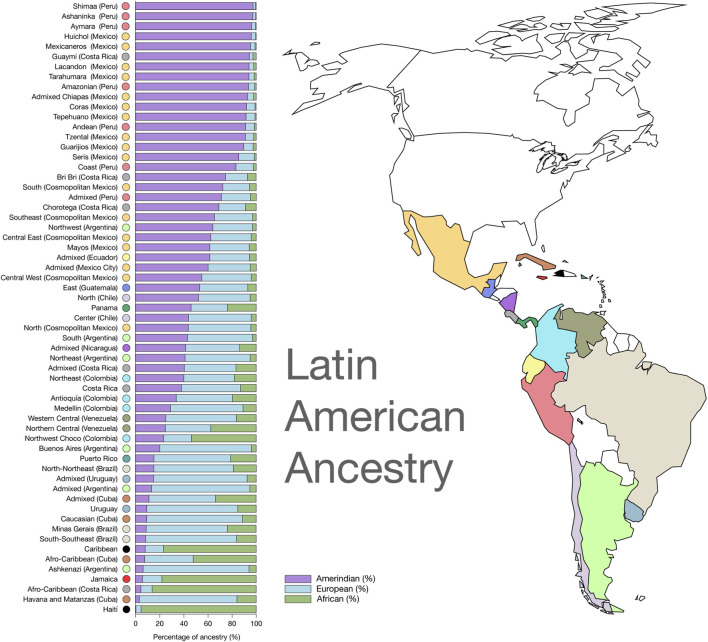
Heterogeneous composition of Latin American ancestry. From Quiñones et al. (2025).

The current genetic makeup of Latin Americans traces back to an initial migration from Northeastern Asia through Beringia approximately 15,000–18,000 years ago (Fagundes et al., 2008; Perego et al., 2009; Reich et al., 2012; Salzano and Sans, 2014; Ruiz-Linares et al., 2014; STATISTA, 2025). Subsequent waves of migration, including European colonization and the trans-Atlantic slave trade, which began in earnest in 1,492, further enriched the genetic and phenotypic diversity of the population (Homburger et al., 2015; Adhikari et al., 2016; Norris et al., 2018). This complex demographic history has led to a high level of genetic heterogeneity, which remains underrepresented in global genomic databases, especially in the southern South American populations. Figure 1 shows the ancestry panorama for the Latin American populations.

Genome-Wide Association Studies (GWAS) have yielded robust results in homogeneous populations but often perform poorly in admixed groups like Latin Americans, potentially producing biased or spurious associations (Belbin et al., 2018; Suarez-Kurtz, 2021). This is particularly critical in pharmacogenomics, where gene-drug interactions can vary substantially by ancestry. Latin America, due to its unique admixture patterns and linkage disequilibrium (LD) structures, serves as a natural laboratory for exploring these relationships.

In admixed Latin American populations, patterns of linkage disequilibrium (LD), haplotype blocks, and variant tagging differ markedly from those in European or East Asian groups. Studies by Homburger et al. (2015), Suarez-Kurtz (2021), and Norris et al. (2018) show that ancestry-driven LD variation can alter pharmacogenetic associations and reduce the predictive accuracy of models derived from homogeneous populations. These differences are especially relevant for genes like *CYP2C9*, *TPMT*, and *CYP2C19*, where LD patterns impact tagging and interpretation. Such complexity underscores the need for guidelines tailored to Latin America’s unique genomic architecture, where standard algorithms may underperform.

Efforts to characterize pharmacogenetic variation in Latin America began in 1984 in Chile with studies of metabolic enzyme polymorphisms among Atacameño individuals (Goedde et al., 1984), followed by research in Amerindian populations from Panama, Uruguay, Chile, Colombia, and Mexico. Later studies included mestizo populations from Cuba, Chile, Costa Rica, and Nicaragua. A major regional initiative, the Ibero-American Network of Pharmacogenetics and Pharmacogenomics (RIBEF), was launched in 2006 under CYTED and has since generated extensive pharmacogene data across Latin America (Quiñones et al., 2025).

In 2019, RELIVAF was established with support from CYTED. With 120 members from 17 countries and territories across Latin America, RELIVAF brings together researchers, academics, clinicians, and regulatory experts to develop population-specific pharmacogenomic guidelines that account for the region’s genetic diversity and admixed ancestry (https://cyted.org/RELIVAF). To date, the network has produced almost a hundred of publications indexed in Web of Science, advancing the regional pharmacogenomics agenda.

Several variants with clinical relevance demonstrate distinct allele frequencies and effect sizes in Latin Americans compared to Europeans. For example, polymorphisms in *NUDT15* (e.g., rs116855232) associated with 6-mercaptopurine toxicity are more frequent in Peru (11.8%), Uruguay (8.8%), and Chile (9.9%) than in European populations (0.2%). Similarly, SNPs in *TPMT* and methotrexate pathway genes (e.g., *MTHFR* and *SLC19A1*) display ethnogeographic distribution patterns linked to ancestral proportions, underscoring the need for ancestry-informed therapeutic strategies (Norris et al., 2018; Moriyama et al., 2016; Soler et al., 2018; Ferreira et al., 2020; Silgado-Guzmán et al., 2022). Additionally, recent studies in the Colombian population have identified significantly ancestry-enriched variants with pharmacogenomic associations across all levels of evidence. Notable examples include ancestry-enriched variants associated with tacrolimus metabolism and methotrexate-induced toxicity. These findings reinforce the observed link between ancestry and health in Latin American populations and underscore the need for collaborative efforts to establish pharmacogenomic profiles across the region (Mariño-Ramírez et al., 2025).

The underperformance of pharmacogenetic algorithms based on European data, such as those for warfarin dosing, in admixed populations like Brazilians and Caribbeans further highlights the need for local validation and model adaptation. Moreover, studies have emphasized that the cumulative frequency of *TPMT* and *NUDT15* risk variants can reach nearly 20% in Latin America, compared to about 5% in European populations (Norris et al., 2018).

In summary, Latin America’s unparalleled genetic diversity presents both a challenge and an opportunity for pharmacogenomic research. The development and implementation of clinical pharmacogenomic protocols must integrate local genetic data and consider the unique admixture and demographic history of the region. Networks like RIBEF and RELIVAF are leading this transformation, fostering the generation of evidence and capacity-building needed to bring precision medicine into clinical practice across Latin America.

## Why Latin America needs region-specific pharmacogenomic clinical guidelines despite the existence of other consortia such as CPIC and DPWG?

While global pharmacogenomic guideline consortia such as the Clinical Pharmacogenetics Implementation Consortium (CPIC) and the Dutch Pharmacogenetics Working Group (DPWG) have made significant strides in translating genetic knowledge into actionable prescribing recommendations (Caudle et al., 2014; Swen et al., 2011), their applicability in admixed and underrepresented populations, such as those in Latin America, remains limited and potentially insufficient.

The genetic architecture of Latin American populations, shaped by recent admixture and complex ancestry, produces unique allele frequencies, haplotypes, and linkage disequilibrium patterns that are underrepresented in the datasets used to develop CPIC and DPWG recommendations, which rely primarily on European and East Asian populations (Suarez-Kurtz, 2021; Bustamante et al., 2011). This limitation reflects not an omission by CPIC or DPWG, but the global lack of pharmacogenomic datasets from populations of non-European and non-East Asian ancestry. Most ancestry-enriched variants common in Latin America simply lack sufficient functional or clinical evidence to be included in guideline evaluations.

Variants such as NUDT15*2 and *3, which are particularly relevant for thiopurine intolerance, illustrate the strong influence of ancestry on allele frequencies. These variants show markedly higher prevalence in East Asian and Latin American populations compared with European populations (Norris et al., 2018; Silgado-Guzmán et al., 2022), underscoring the importance of considering local ancestry composition when interpreting pharmacogenomic test results.

In contrast, warfarin provides a separate and well-established example in which pharmacogenetic models developed in European or East Asian populations often perform poorly in admixed Latin American and Caribbean populations. These differences arise from distinct allele-frequency patterns, alternative functional variants, and unique linkage disequilibrium structures, highlighting the need to evaluate each gene–drug pair individually rather than assuming direct transferability across ancestries (Suarez-Kurtz and Botton, 2015; Galvez et al., 2018).

Marked differences in allele frequencies across Latin American populations limit the applicability of international guidelines in the region. Variants enriched in Indigenous, African, or admixed populations remain poorly characterized due to their underrepresentation in global datasets, a challenge shared by many non-European groups. RELIVAF acknowledges these limitations and aims to: (i) make such gaps visible in a regional framework; (ii) integrate all available Latin American evidence, including Spanish- and Portuguese-language studies; (iii) provide context-adapted recommendations even when data are limited; and (iv) prioritize under-studied alleles for multicenter validation. While CPIC and DPWG offer more mature curation models, RELIVAF seeks to progressively emulate this approach. This first regional guideline aims to catalyze curated databases and coordinated evidence generation across Latin America.

Including studies published in Spanish and Portuguese can partially mitigate information gaps for Latin American populations. However, expanding the search to these sources cannot necessarily compensate for the fundamental lack of large, high-quality studies involving underrepresented populations, which remains the core limitation.

International pharmacogenomic guidelines often overlook Latin America’s clinical, socioeconomic, and infrastructural realities, including limited testing access and variable standards of care (Cacabelos et al., 2021). RELIVAF addresses this by incorporating regional pharmacological interactions and, when relevant, dietary habits, particularly for anticoagulants. Its framework balances clinical validity with implementation feasibility by aligning recommendations with national capacities and data availability (Weitzel et al., 2014). RELIVAF also documents country-specific interaction profiles and nutritional patterns that influence drug response, ensuring context-appropriate prescribing guidance.

The creation of Latin America-specific pharmacogenomic clinical guidelines would ensure that:

1. Locally relevant genetic variants and allele frequencies are incorporated.
2. Recommendations are adapted to regional therapeutic protocols and formularies.
3. The guidelines are harmonized with local regulations, testing capabilities, and healthcare models and economics, and are culturally sensitive.
4. Underrepresented groups, particularly the Afro-Latin and Indigenous populations, are considered in risk stratification and therapeutic decision-making.

While CPIC provides recommendations for alleles with available evidence, RELIVAF adds essential regional granularity for Latin America. Allele frequencies are reported by country and, when possible, by ancestry cluster, enabling identification of variants that are globally rare but locally common. Some Latin American regions also exhibit consanguinity, which can increase homozygosity and enrich rare alleles, while Indigenous populations often show high frequencies of population-specific variants in ADME genes due to historical isolation (De Castro and Restrepo, 2015). RELIVAF aligns recommendations with national drug availability and clinical protocols to ensure feasibility, highlights regionally relevant alleles often absent from commercial panels, and systematically identifies evidence gaps. In doing so, RELIVAF complements, rather than duplicates, the foundational work of CPIC and DPWG.

The *Red Latinoamericana de Implementación y Validación de Guías Clínicas Farmacogenéticas* (RELIVAF) has already demonstrated the feasibility and value of this regional approach, promoting evidence generation, capacity building, and collaborative implementation of population-tailored pharmacogenomic practices.

In conclusion, while CPIC and DPWG provide essential foundational knowledge, the development of Latin American pharmacogenomic clinical guidelines is imperative to ensure equity, accuracy, and feasibility in the application of precision medicine in this highly diverse region.

As an initial strategy, RELIVAF has prioritized the development of three pharmacogenomic clinical guidelines:

1. *DPYD* genotyping for fluoropyrimidine-based chemotherapy
2. Genotype-guided dosing of coumarin anticoagulants, and
3. *TPMT* and *NUDT15* genotyping for purine-based treatment with focus on paediatric patients.

This selection is based not only on the clinical relevance of these pharmacogenomic interventions, both associated with widely used, high-risk medications, but also on their documented cost-effectiveness, existing evidence of clinical utility, and the practical feasibility of implementation within the regulatory, infrastructural, and economic frameworks of several Latin American countries. Furthermore, these cases exemplify the disparities in drug safety and access across the region, reinforcing the urgent need for region-specific guidance and standardized practice.

The *DPYD* gene, which encodes dihydropyrimidine dehydrogenase (DPD), plays a critical role in the metabolism of fluoropyrimidines such as 5-fluorouracil, capecitabine and tegafur. Carriers of reduced-function *DPYD* alleles are at high risk for life-threatening toxicities when treated with standard fluoropyrimidine doses. Although drug labels and certain regulatory or clinical bodies strongly recommend pre-treatment *DPYD* genotyping, CPIC and DPWG do not issue recommendations about whether testing should be performed; rather, they provide guidance on how to adjust therapy once genotype information is available. Despite these recommendations, the clinical use of *DPYD* genotyping in Latin America remains minimal. This is particularly concerning given that the distribution of pathogenic *DPYD* variants appears to vary across populations, and Latin America, being highly admixed, likely harbours unique or under-characterized alleles. Moreover, the region suffers from serious underreporting of adverse drug reactions, especially in oncology, making proactive genotyping even more critical for patient safety. Several clinically relevant *DPYD* variants have not been systematically evaluated in Latin American populations, despite their potential to cause severe or fatal toxicities. Beyond the well-established actionable variants recognized by CPIC, rs3918290 (DPYD*2A), rs55886062 (c.1679T>G, DPYD*13), rs75017182 (c.1236G>A/HapB3) and rs67376798 (c.2846A>T, DPYD*9B), other alleles with elevated frequencies in the region, such as rs2297595 (c.1601G>A) in LD with rs1801625, may also have significant functional consequences. The presence of such variants underscores the ethical and legal imperative to incorporate *DPYD* genotyping into clinical oncology practice across Latin America, to prevent avoidable toxicity and ensure equitable patient protection (Hamzic et al., 2021; Cerpa, 2024; Quiñones, 2025).

Although some effect size estimates are available from regional cohorts, such as the contribution of *NUDT15* to thiopurine intolerance or *DPYD* variants to fluoropyrimidine toxicity, many Latin American populations remain underrepresented in clinical studies. This lack of locally derived effect-size data limits the certainty of pharmacogenomic associations and their applicability across the region. To address this, RELIVAF applies the GRADE framework to qualify recommendations and transparently communicate uncertainty, while also identifying critical gaps for future validation studies.

RELIVAF will develop a clinical guideline for genotype-guided dosing of coumarin anticoagulants, warfarin, acenocoumarol, and phenprocoumon, which are widely used across Latin America for thromboembolic prevention. Warfarin predominates in public health systems, while acenocoumarol is common in Chile and parts of Argentina, Uruguay, and Brazil. The pharmacogenetic relevance of these agents is well established: variants in *CYP2C9*, *VKORC1*, and *CYP4F2* influence dose variability and bleeding risk, and *NQO12* (rs1800566) may further modulate dosing in Latino populations (Bress et al., 2012; Duconge et al., 2016; Hernandez-Suarez et al., 2016; El Rouby et al., 2021)*.* In Chile, a locally developed algorithm combining *VKORC1, CYP2C9*2/*3, and clinical variables explained nearly 50% of acenocoumarol dose variability (Roco et al., 2020), differing from European models. These results highlight the value of region-specific pharmacogenetic tools. Supporting this, a recent survey across 17 countries ranked CYP2C9/VKORC1-coumarins among the highest-priority gene-drug pairs (96%–99%) for clinical implementation (Salas-Hernández et al., 2023), reflecting broad professional consensus.

Thiopurines (6-mercaptopurine, thioguanine, azathioprine) are cornerstone agents in pediatric acute lymphoblastic leukaemia (ALL), but their narrow therapeutic index makes early myelotoxicity a frequent cause of treatment interruption. Loss-of-function variants in *TPMT* and *NUDT15* are well-established predictors of intolerance, and CPIC and DPWG provide strong, phenotype-stratified dosing recommendations (Relling et al., 2019). However, these guidelines were developed in European and North American populations, assuming allele frequencies and healthcare infrastructure not representative of Latin America. In Chile and neighbouring countries, Amerindian-European admixture elevates *NUDT15* c.415C>T frequencies, with minor allele frequencies (MAF) around 7%–8%, including ∼12–13% heterozygotes and 1%–2% homozygotes, substantially higher than in Europe (<1%) (von Muhlenbrock et al., 2023). This confers a significantly higher baseline risk of leukopenia and neutropenia at standard 6-MP doses. In contrast, *TPMT* loss-of-function alleles occur at ∼8%, mainly TPMT*3A, resembling Caucasian profiles. Thus, *NUDT15* is a more relevant predictor of toxicity in this region (Farfan et al., 2014; Alvarez et al., 2009; Garrido et al., 2013). Additionally, many Latin American centres lack routine therapeutic drug monitoring (TGN/TiMe) and infrastructure for close dose adjustments, making pre-emptive genotype-guided dosing especially valuable. Conservative initial dosing in carriers of NUDT15*1/*3 or biallelic variants can significantly reduce hospitalizations and treatment interruptions (Dean et al., 2012). From a health-economic perspective, where *NUDT15* prevalence and adverse event costs are high, pre-treatment genotyping with stronger dose-reduction thresholds is more cost-effective than applying unmodified European or U.S.-based protocols, where the utility of *NUDT15* testing is less clear (Roberts et al., 2025).

In this context, the integration of *TPMT* + *NUDT15* testing into routine ALL paediatric management represents not only a precision-medicine imperative but also a pragmatic, regionally justified adaptation. Using CPIC as the scientific foundation while tailoring implementation to Latin American allele frequencies, drug availability, and monitoring capacity offers the most rational path to reducing toxicity, improving treatment continuity, and optimizing resource use across the region.

In summary, these three topics were selected due to their high clinical impact and mortality risk if unmanaged, the presence of established pharmacogenomic evidence in the region, the regional variability in allele frequencies and drug usage, and the need for guidance in public health settings, where implementation is most challenging and most needed.

By starting with *DPYD*, *TPMT* + *NUDT15*, and coumarin-related genes, RELIVAF aims to deliver high-priority, high-impact pharmacogenomic tools that can be feasibly adopted across Latin American healthcare systems and that exemplify the importance of locally adapted, evidence-based clinical guidance.

## RELIVAF guideline development process

RELIVAF pharmacogenomic guidelines are based on international frameworks such as CPIC and DPWG, adapted to meet Latin American needs. CPIC recommendations provide the foundational evidence base, while RELIVAF introduces region-specific adaptations, including alignment with national formularies, stratification by ancestry clusters, adjustments for laboratory infrastructure, and consideration of linguistic, regulatory, and reimbursement barriers. The development process emphasizes clinical feasibility across diverse healthcare settings, supported by systematic evidence reviews and regionally relevant data, such as allele frequencies and therapeutic availability, to ensure scientific rigor, actionability, and equitable implementation.

**FIGURE 2:**
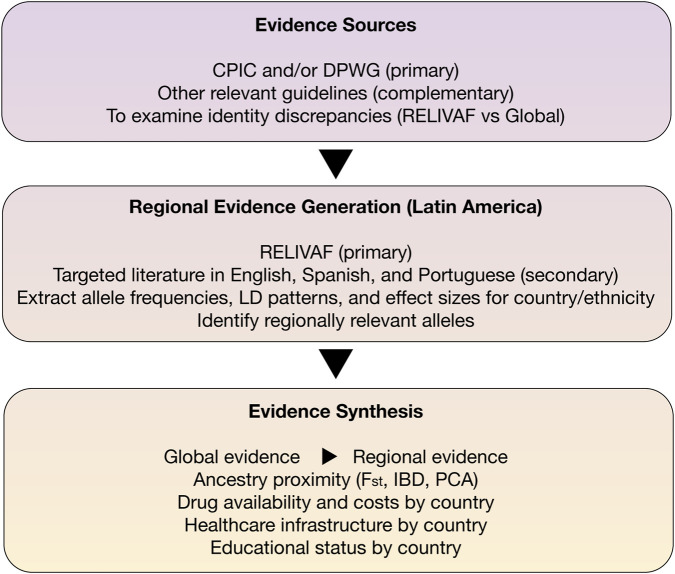
Three-layer evidence review and integration workflow. *F*_*st*_ *= Fixation Index, IBD = (Identity By Descent), PCA = Principal Component Analysis*.

To clarify the RELIVAF guideline-development process, we adopt a three-layer evidence integration strategy. First, CPIC and DPWG reviews serve as the primary evidence base. Second, RELIVAF conducts complementary searches for Latin American studies, including publications in English, Spanish, and Portuguese. Third, contextual factors, such as ancestry-enriched allele frequencies, local drug formularies, and implementation feasibility, are systematically incorporated (Figure 2). All evidence is evaluated using a unified GRADE framework. Greater weight is given to high-quality peer-reviewed studies (Q1-Q2), while data from non-curated sources or genotyping methods with limited validation are transparently identified. This approach balances scientific rigor with the need to include all relevant regional data.

Each RELIVAF guideline will explicitly include a section describing clinically relevant pharmacological interactions frequently observed in Latin American settings, considering both polypharmacy patterns and country-specific therapeutic protocols. In addition, dietary and nutritional habits characteristic of each subregion, such as high vitamin K intake from local vegetables influencing anticoagulant response, or folate and methionine consumption affecting fluoropyrimidine metabolism, will be evaluated as potential modulators of genotype-to-phenotype relationships. These culturally dependent factors are crucial to accurately predict treatment response and to contextualize recommendations for coumarin anticoagulants and other drugs with narrow therapeutic windows.

Each guideline will outline the pharmacogenomic relevance of the gene–drug pair, associated clinical risks, and recommended actions. Recommendations will include evidence grading and a clear rationale based on both global and regional data. Sources will be cited and, when appropriate, supported by prescribing tables. Identified evidence gaps will guide future regional research and implementation priorities.

The pharmacogenetic background will describe gene function, key polymorphisms, and allele/genotype frequencies disaggregated by country and ancestry, supplemented by global references (e.g., 1,000 Genomes, gnomAD). Analytical reliability will be ensured through participation in proficiency testing schemes for genotyping. Interpretation of genetic tests will follow standardized genotype-to-phenotype translations (e.g., poor, intermediate, normal, ultrarapid metabolizers), with technical guidance on validated testing platforms and coverage. Sample reports and genotype–phenotype tables will support harmonized clinical interpretation across laboratories.

Therapeutic recommendations will be stratified by phenotype and graded using the GRADE methodology (Balshem et al., 2011), with at least one actionable intervention per phenotype (e.g., dose adjustment or alternative drug selection), as recommended by Guyatt et al. (2008). The GRADE framework will be applied to both global and regional evidence. When Latin American data are sparse, CPIC and DPWG evaluations will be incorporated as high-quality external evidence, and any regional studies, when available, will be assessed separately. Summaries will include decision algorithms and prescribing tables adapted to the availability of therapeutic alternatives and monitoring infrastructure in different Latin American countries. In situations where overall evidence remains limited, RELIVAF will issue conditional or weak recommendations with explicit statements describing the underlying uncertainty. This integrated approach underscores the need for prospective validation studies in Latin American populations (Cavallari et al., 2019).

Pharmacist-centred recommendations will define structured follow-up protocols, including frequency of monitoring, clinical endpoints, and use of validated tools for adherence and toxicity assessment (Tommelein et al., 2014). Ethical and operational considerations require careful integration of technical feasibility with sociopolitical determinants, such as public health priorities, healthcare infrastructure, and equitable access to genetic services (Bonham et al., 2018). This includes addressing regulatory variability and best practices in IRB engagement, while supporting risk-based implementation strategies, such as adapting informed consent requirements for low-risk scenarios to avoid unnecessary barriers to care (Ando and Terada, 2023).

When relevant, pharmacokinetic data, including clearance, half-life, and exposure variability, will be incorporated, with special attention to model-informed precision dosing and available population-specific PK data. Genomic literacy challenges will be addressed through tailored educational resources for clinicians, laboratory professionals, and patients. Guidelines will promote implementation via infographics, institutional protocols, and integration into clinical decision support systems and electronic health records.

Evidence reviews will include publications in English, Spanish, and Portuguese, drawing from PubMed, Embase, SciELO, LILACS, and LatinDex. This strategy enables the inclusion of Latin American pharmacogenomic studies that, while peer-reviewed, may not be indexed in international databases and are thus often excluded from global reviews. Examples include national studies on *TPMT*, *CYP2C9*, and *CYP2D6* variants from Chile, Colombia, and Uruguay. Strength of evidence and recommendation grade will follow the GRADE framework (Balshem et al., 2011).

The prioritization of gene-drug pairs will consider: (1) perceived clinical relevance based on surveys ranking 54 gene–drug pairs by Latin American professionals (Salas-Hernández et al., 2023); (2) polymorphisms with wide population coverage across countries; and (3) the presence of regional clinical validation studies, including efficacy, toxicity, and pharmacokinetic outcomes.

Guideline development will be coordinated by a multidisciplinary steering committee of pharmacogenomics experts, clinicians, pharmacists, bioethicists, and laboratory professionals, selected through an open call within RELIVAF. Participation will be documented, and all members must disclose personal and financial conflicts of interest (COI), including those of immediate relatives.

Once approved by RELIVAF’s steering committee, each guideline topic will be assigned to a multidisciplinary writing team led by one or two chairs. These experts will conduct literature reviews and draft recommendations following IOM standards, with support from scientific curators. One or two scientific curators will support each team, providing expertise in evidence curation, genotype-phenotype mapping, actionability assessment, and preparation of background materials and tables. Curators will also monitor new findings and recommend updates when necessary. All recommendations will follow a clear, actionable format specifying the intervention and conditions for its use. Draft guidelines will undergo open review by RELIVAF members and external stakeholders.

Accordingly, validation will include an internal assessment using the AGREE II instrument (AGREE II Consortium, 2009) and external peer review. Each guideline will be submitted to a Q1–Q2 peer-reviewed journal, while its regional open-access dissemination in English, Spanish, and Portuguese will take place through RELIVAF, SOLFAGEM, regional scientific societies, and the Latin American Pharmacogenomics Congress. External validation will involve international curator-reviewers from recognized pharmacogenomic consortia (e.g., CPIC, DPWG) to ensure methodological rigor, scientific oversight, and harmonization with global standards. This external review will also include representatives spanning the full spectrum of relevant perspectives: clinical experts, professional organizations and scientific societies, regulatory agencies, patients or patient advocates, and members of the public.

Guidelines will be reviewed every 2 years to incorporate new evidence, with comprehensive updates every 5 years. A defined procedure will allow interim revisions to ensure continued scientific validity and clinical relevance across countries in the region.

Based on the above, the proposed structure and content of the RELIVAF guidelines are outlined in Table 1. A general illustrative framework for genotype–phenotype translation is summarized in Table 2.

**TABLE 1:** Structure and Content of the RELIVAF Pharmacogenomic Clinical Guidelines (standard format).

Section	Content description
1. Background and Clinical Relevance	• Overview of the pharmacogenomic importance of the selected gene–drug pair • Mechanisms by which genetic variation influences drug response (e.g., metabolism, transport, target binding) • Clinical implications of gene–drug interactions (e.g., toxicity, treatment failure) • Relevance to Latin American clinical practice (e.g., disease burden, usage patterns in public health systems) • Rationale for prioritization by RELIVAF.
2. Pharmacogenetic Background	• Gene function and biological role • Description of clinically relevant polymorphisms • Integrated allele and genotype frequencies from Latin American populations, disaggregated by country and ancestry, and compared with global reference datasets (e.g., 1,000 Genomes, gnomAD)
3. Genetic Test Interpretation	• Standardized genotype-to-phenotype translation (e.g., poor, intermediate, normal, ultrarapid metabolizers) • Sample genotype reports and interpretative guidance for clinical laboratories • Technical considerations: recommended testing platforms and coverage (e.g., SNP panels, targeted sequencing) • Proficiency testing for genotyping, ensuring the analytical accuracy and reliability • Genotype–Phenotype Table summarizing expected clinical phenotypes
4. Therapeutic Recommendations	• Actionable prescribing recommendations by phenotype: - Dose adjustment - Drug substitution or avoidance - Additional monitoring requirements • Summary table of recommendations across phenotypes is required • Decision-making algorithms or flowcharts for clinical application • Discussion of applicability based on drug availability and monitoring resources across Latin American countries
5. Pharmacist and Clinical Care Recommendations	• Roles and responsibilities of clinical pharmacists and prescribing clinicians • Suggested monitoring plans: - Follow-up strategies (e.g., laboratory tests, clinical assessments) - Tools for toxicity and efficacy monitoring (e.g., INR, blood counts) - Approaches to assess medication adherence • Key points for patient education and counselling
6. Ethical, Regulatory, and Operational Considerations	• Overview of ethical and administrative heterogeneity across Latin America regarding genetic testing • Recommendations for low-risk implementation, including support for not requiring written informed consent for routine pharmacogenomic testing (Ando and Terada, 2023) • Examples of flexible Institutional Review Board policies and national regulatory frameworks • Best practices for sample collection, biobanking, and genetic data governance
7. Pharmacokinetic Considerations (if applicable)	• Genotype effects on key pharmacokinetic parameters (e.g., clearance, half-life, C_max_) • Population pharmacokinetic data from Latin American cohorts (if available) • Guidance on the use of model-informed precision dosing when relevant
8. Pharmacogenomic Literacy and Implementation Strategies	• Challenges related to genomic literacy in the region • Educational recommendations for: - Healthcare providers (undergraduate and postgraduate levels) - Clinical laboratory professionals - Patients and communities • Implementation tools: - Infographics, case studies, institutional protocols - Integration into electronic health records (EHRs) and clinical decision support systems (CDSS)
9. Evidence Review and Grading	• Methodological approach for literature selection: - Inclusion of sources in English, Spanish, and Portuguese - Databases: PubMed, SciELO, Embase • Types of evidence considered (RCTs, meta-analyses, observational studies, *in vitro*/*in silico* studies) • Application of the GRADE framework to rate evidence quality and recommendation strength
10. Knowledge Gaps, Limitations, and Research Priorities	• Identification of gaps in current data: - Allele frequencies in underrepresented countries or ethnic groups - Clinical outcome data in Latin American populations - Local pharmacokinetic/pharmacodynamic validation studies • Recognition of limitations in generalizability • Recommendations for future research in the region
11. Applicability by Ethnic and National Groups	• Analysis of genetic proximity among ethnic groups by country to support extrapolation of dosing recommendations • Evaluation of drug availability by country and ethnic distribution • Identification of political and administrative barriers to implementation and strategies to address them
12. Guideline Development Process	• Composition of the writing group and expert reviewers • Criteria for selecting gene–drug pairs and country participation • Declaration and management of conflicts of interest (COIs) • Adherence to established methodological standards (e.g., AGREE II instrument)
13. Updating Policy	• Annual review of emerging scientific evidence • Full guidelines update every 5 years • Process for proposing and validating interim modifications
14. Dissemination Plan	• Open-access dissemination through RELIVAF and SOLFAGEM platforms • Trilingual summaries in English, Spanish, and Portuguese • Promotion through professional societies, scientific congresses, and national clinical networks • Public access to educational resources and implementation tools
15. References and Supplementary Materials	• Comprehensive reference list • Supplementary tables: - Allele frequencies by country and ethnicity - GRADE evidence classifications - Conflict of interest declarations

**TABLE 2:** Illustrative conceptual framework for understanding genotype–phenotype translation.

Generic genotype pattern (illustrative only)^a^	Predicted phenotype	Enzyme/Transporter activity or function	Clinical implication^b^	Recommended clinical action^c^
Normal/Normal	Normal Metabolizer	Normal activity	Expected response at standard dose	Use standard dosing
Normal/Reduced	Intermediate Metabolizer	Decreased activity	Possibly reduced efficacy or altered exposure	Consider dose adjustment or closer monitoring
Reduced/Reduced	Poor Metabolizer	Low or no activity	Increased risk of toxicity or reduced drug clearance	Avoid the drug or reduce the dose; choose an alternative
Normal/Increased	Rapid Metabolizer	Increased activity	Reduced drug levels, risk of treatment failure	Consider a dose increase or alternative therapy
Increased/Increased	Ultrarapid Metabolizer	Significantly increased activity	Therapeutic failure due to low drug levels	Avoid drug use or use a higher dose if supported by evidence
No function/No function	Non-functional	No activity	Severe loss of function; the drug may be ineffective or unsafe	Contraindicated or use an alternative
Unknown/Unknown	Indeterminate Phenotype	Unknown or variable	Unclear clinical implications	Use clinical judgment; monitor closely
Example 1^d^	​	​	​	​
*CYP2D6* ^*^1/^*^1	Normal Metabolizer	Normal enzyme activity	Expected therapeutic response at standard dose	Prescribe standard dose for drugs metabolized by *CYP2D6* (e.g., fluoxetine)
Example 2^d^	​	​	​	​
*CYP2C19* ^*^2/^*^2	Poor Metabolizer	Low or no activity	Increased risk of toxicity or reduced drug clearance	Avoid drug or reduce dose; choose alternative (e.g., clopidogrel)

^a^ Allele Function Definitions:

Normal: Comparable enzymatic activity to the wild-type allele.

Reduced: Partial loss of function relative to wild-type.

No function: Complete loss of activity due to deleterious variants (e.g., nonsense, frameshift).

Increased: Enhanced activity (e.g., gene duplication, promoter gain).

Uncertain: Insufficient evidence to classify function.

^b^ Drug response may vary depending on whether the agent is a prodrug activated by metabolism.

^c^ Variant frequencies vary across Latin American populations; some genotypes may be rare or absent in specific groups. Clinical recommendations should consider local allele distributions.

^d^ These examples are for illustrative purposes and aligned with CPIC definitions.

RELIVAF, adopts CPIC/DPWG, phenotype definitions for each gene–drug pair unless new, validated evidence supports modification.

To ensure consistency in clinical implementation, RELIVAF will support the development of interoperable digital infrastructure for pharmacogenomic data. Although no single platform is currently recommended, the guidelines advocate for the integration of genotype information into national electronic health records (EHRs) and clinical decision support systems (CDSS), enabling secure and longitudinal access across healthcare facilities. RELIVAF also will encourage alignment with emerging digital health initiatives in Latin America, fostering regional harmonization and data portability.

## Latin American-specific extensions to CPIC and DPWG guidelines

Although the RELIVAF guidelines will adopt a structure compatible with internationally recognized frameworks such as those of the CPIC and the DPWG, they introduce several key distinctions as sections that reflect the unique genetic, clinical, economic, and educational realities of Latin America.

**FIGURE 3:**
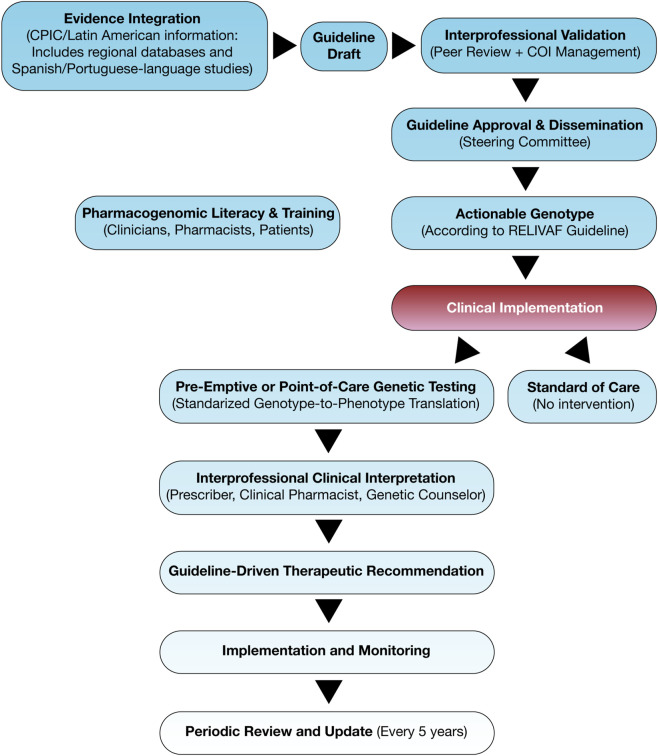
RELIVAF Guideline Development and Pharmacogenomic Implementation workflow.

First, RELIVAF incorporates a culturally sensitive strategy to promote pharmacogenomic literacy, recognizing that foundational knowledge is limited across Latin America, even among biomedical professionals. Unlike CPIC and DPWG, which assume baseline familiarity with genetic testing, RELIVAF addresses the educational gap through clinician, pharmacist, and laboratory training, as well as patient- and public-facing initiatives. To support these efforts, a modified Spanish- and Portuguese-language version of the MAPL software (Allen et al., 2022) will be deployed, adapted to local formularies, infrastructure, and regulatory settings. RELIVAF promotes an interprofessional implementation model in which prescribers, pharmacists, laboratory personnel, and genetic counselors collaborate across the pharmacogenomic care continuum, from test ordering and interpretation to therapy adjustment and patient counseling. This collaborative framework will be reflected in standardized clinical protocols and role-based implementation strategies across healthcare facilities. RELIVAF follows a structured, interprofessional workflow for guideline development and implementation, incorporating international evidence, regional data, COI safeguards, and educational strategies. Figure 3 outlines this process, from evidence review through genotype–phenotype translation to context-adapted clinical application across Latin American health systems.

Second, RELIVAF considers the dynamic landscape of drug availability and regulatory approval across Latin American countries. Many genotype-guided therapies included in CPIC/DPWG guidelines are unavailable, inconsistently distributed, or subject to restrictions in the region. A representative example is the CPIC and DPWG recommendation to switch from clopidogrel to prasugrel or ticagrelor in *CYP2C19* poor or intermediate metabolizers. However, in many Latin American public healthcare systems, these alternatives are not included in national formularies, are inconsistently available, or are cost-prohibitive, leaving clopidogrel as the only feasible option. Such limitations constrain the implementation of genotype-guided therapy. For instance, Brazil’s public system (SUS) has historically covered only clopidogrel, while other countries restrict prasugrel or ticagrelor to privately insured patients or select profiles (Muntaner et al., 2021; Athanazio et al., 2022). More broadly, Persaud et al. (2019) found marked differences in essential medicines lists across 137 countries, including Latin America. These disparities illustrate the structural and economic barriers that limit adherence to pharmacogenomic guidelines. A recent clinical guideline from the United Kingdom Centre for Pharmacogenomics and Stratified Medicine (Dello Russo et al., 2025) reaffirms the validity of *CYP2C19*-guided clopidogrel prescribing, while emphasizing that implementation feasibility depends on drug availability, an especially relevant consideration in Latin America. RELIVAF addresses this by prioritizing gene–drug pairs with confirmed access in national formularies to ensure clinical actionability.

Third, RELIVAF emphasizes the inclusion of locally generated evidence, including studies published in Spanish and Portuguese that may not be captured by international reviews such as those by CPIC or DPWG. While not all of these studies meet the strictest quality thresholds (e.g., randomized trials or Q1 journals), many are nonetheless relevant for regional pharmacogenomic contexts. This approach enhances regional scientific visibility and ensures that recommendations reflect local allele frequencies, LD patterns, heterogeneity in effect size estimates, prescribing practices, and ancestry-informed patterns. When available, data will be stratified by ancestry to identify knowledge gaps and research priorities.

Fourth, RELIVAF addresses the regulatory and ethical heterogeneity of pharmacogenomic implementation in the region. Clinical research and sample collection procedures vary widely due to differing national laws, ethics board requirements, and genetic data policies. These considerations are central to RELIVAF’s approach, particularly to avoid perpetuating disparities affecting historically underrepresented groups, such as Indigenous and Afro-descendant populations (Popejoy and Fullerton, 2016).

Given these differences, RELIVAF guidelines will address ethical, literacy, and regulatory feasibility on a country-by-country basis, recognizing the importance of adapting implementation strategies to the local context. This approach is essential to facilitate responsible and practical adoption of pharmacogenomic testing within national healthcare systems, particularly in countries where infrastructure or regulation is still evolving.

In all cases, each document will strongly recommend that written informed consent should not be considered mandatory for pharmacogenomic testing conducted as part of routine clinical care, particularly when it is integrated into standard diagnostic procedures and not associated with research protocols. This recommendation aligns with the position of Ando and Terada (2023), who advocate for a pragmatic and risk-adapted approach to consent in clinical pharmacogenomics, especially in settings where excessive procedural barriers could limit patient access and delay the benefits of personalized therapy.

RELIVAF therefore promotes a framework that balances ethical rigor with clinical accessibility, ensuring that genomic medicine can be equitably implemented while respecting national norms and minimizing unnecessary barriers to care.

To support the cross-national applicability of its pharmacogenomic guidelines, RELIVAF will use population genetics tools to evaluate genetic relatedness among Latin American populations. Methods such as principal component analysis (PCA), admixture-based ancestry estimation, F_ST_ genetic distance metrics (Fixation Index), and haplotype structure analysis will be applied to assess inter-population similarities and differences.

Allele frequency data will be drawn from global resources, including the 1000 Genomes Project, gnomAD, and The Cancer Genome Atlas (TCGA), as well as from local studies conducted by RELIVAF collaborators. Additional data will be integrated from emerging regional initiatives such as LatinGen (http://www.latingen.org), the Latin American Genomic Consortium (https://www.latinamericangenomicsconsortium.org), LatinGenomes (https://x.com/latingenomes?lang=es), BIPMed (https://www.bipmed.org), and ChileGenomico (https://chilegenomico.med.uchile.cl/home/).

By combining these data sources and analytical approaches, RELIVAF aims to identify clusters of populations with shared genomic profiles. This will support the careful generalization of gene–drug recommendations across countries, while maintaining relevance to local variation.

For instance, the 1000 Genomes Project has shown that Peruvians from Lima (PEL), Mexicans of Los Angeles (MXL), and Colombians from Medellín (CLM) share substantial Native American and European ancestry, with variable African contributions (1000 Genomes Project Consortium et al., 2015). These shared patterns suggest that clinically relevant allele frequencies, such as TPMT*3C (rs1142345) or CYP2C9*2/*3, may be comparable across these populations. As a result, harmonized recommendations for thiopurines or warfarin dosing may be both feasible and justified in these settings.

RELIVAF aims to deliver regionally coordinated pharmacogenomic guidelines while allowing country-specific flexibility based on drug availability, healthcare infrastructure, and regulatory contexts. Its framework incorporates elements rarely addressed by CPIC or DPWG, including a pharmacist-centered care model focused on therapeutic follow-up, safety monitoring, and individualized care pathways. Additionally, RELIVAF provides pharmacokinetic adjustment tools to support dosing decisions informed by local data. The initiative aligns with the National Academy of Medicine (NAM/IOM) standards for trustworthy guideline development, emphasizing methodological transparency, external peer review, scheduled updates, conflict-of-interest disclosure, and stakeholder inclusion. Beyond these principles, RELIVAF introduces ancestry-stratified recommendations and context-sensitive implementation strategies tailored to the diverse healthcare realities of Latin America.

In line with these standards, conflicts of interest (COIs) will be managed through mandatory annual disclosures, with contributors required to recuse themselves from deliberations or voting when direct financial, academic, or intellectual conflicts are present. Each guideline will also undergo independent external review to ensure methodological transparency and minimize potential bias.

In summary, RELIVAF distinguishes itself by promoting genomic literacy, addressing drug availability disparities, and prioritizing Latin American evidence. This approach ensures that pharmacogenomic guidelines are scientifically robust, regionally relevant, and clinically actionable.

## Conclusion

Latin America presents a unique opportunity to advance pharmacogenomics, given its rich genetic diversity and the urgent need for tailored therapeutic approaches. Yet, global guidelines often fail to account for the region’s specific allele frequencies, patterns of admixture, and healthcare disparities. In response, RELIVAF is developing population-specific pharmacogenomic guidelines that integrate international standards with regional evidence, drug availability, and implementation realities. These guidelines aim to expand access to precision medicine, improve clinical outcomes, and strengthen local research and regulatory capacity. They are designed to inform, but not replace, clinical judgment, recognizing that real-world variation in drug response may arise even in genetically “normal” individuals.
